# Cancer Research in Adenocarcinoma, Adenoma, Adenomatous Polyposis Coli, and Colitis-Associated Neoplasia: A Special Issue

**DOI:** 10.3390/cancers15041328

**Published:** 2023-02-20

**Authors:** Kentaro Moriichi, Mikihiro Fujiya

**Affiliations:** Gastroenterology and Endoscopy, Division of Metabolism and Biosystemic Science, Gastroenterology, and Hematology/Oncology, Department of Medicine, Asahikawa Medical University, 2-1-1-1 Midorigaoka-Higashi, Asahikawa 078-8510, Hokkaido, Japan

Recent technological advancements have enabled us to analyze a variety of aspects of colorectal cancer (CRC), including both clinical and basic science. It has been revealed that CRC mainly arises from three different pathways, namely, the canonical adenoma-carcinoma sequence, serrated pathway, and alternative pathway [1]. Epigenetic alterations and phenotypes, such as CpG island methylator phenotype [2], also play vital roles in CRC development. Furthermore, multiple omics technologies, such as genomics, transcriptomics, proteomics, and metabolomics, among others, have been applied for the exploration of CRC mechanisms. 

A comprehensive gene analysis of CRC was conducted by The Cancer Genome Atlas (TCGA) project and revealed that CRC can be stratified into two classes, hypermutated and non-hypermutated tumors [3]. Moreover, four consensus molecular subtypes (CMSs) were established to analyze large-scale data, including the TCGA data [4]. Of the four subtypes, CMS4 (mesenchymal, 23%), characterized as epithelial–mesenchymal-transition-related gene expression, is reported to have a poor prognosis. Furthermore, various factors, including non-coding RNA and related substances, the gut microbiome, and altered immune systems [5], are understood to be involved in the carcinogenesis of CRC. In this Special Issue, Konishi et al. and Thomas et al. describe the mechanism of CRC development. Konishi et al. focus on RNA-binding proteins (RBPs). In their study, they performed functional RBP screening using cell lines including the CRC cell line and identified 12 major tumor-associated RBPs which exhibited strong tumor proliferative effects, with no marked changes in expression. These RBPs are expected to be used as therapeutic targets [6]. Thomas et al. reviewed age-related T-cell dysfunction in elderly patients with CRC, including the mechanism of CRC development mediated by aged T-cells. Immune system alterations occur secondary to aging, and T-cells are especially predisposed to aging, since they are constantly subjected to various antigens during an individual lifetime. The importance of T-cell responses to CRC has been highlighted in many studies. The immune system’s impairment due to aging significantly impacts individuals with CRC. Thus, to improve the treatment of elderly patients with CRC, understanding the functional role of senescent T-cells is crucial [7].

Colitis-associated CRC arises from chronic mucosal inflammation in patients with inflammatory bowel disease (IBD). Approximately 15% of IBD patients die due to colitis-associated CRC [8]. Hence, it is important to elucidate this mechanism. In this Special Issue, Sakai et al. investigate gene expression in a mouse colitis-associated CRC model (DSS/AOM-induced enteritis/carcinogenesis mouse model) and fecal microbes. They revealed an altered metabolic pathway, enriched sphingolipid signaling, and lipoarabinomannan biosynthesis, which suggested an interaction between the PI3K-Akt-mTOR pathway and LAM synthesis in colitis-induced carcinogenesis [9].

For CRC screening, the fecal occult blood test (FOBT) and fecal immunochemical test (FIT) have been efficacious. Although FOBT/FIT are very effective tests, the pitfalls of these procedures, namely, the high rate of false positives and false negatives observed in FIT, should be considered. As reported in this Special Issue, Gies et al. evaluated nine different FIT brands and revealed the FIT characteristics stratified by sex and age. They found that a negative FIT was less reliable in ruling out advanced colorectal neoplasia among men than women and among older adults than younger participants [10]. Colonoscopy for CRC screening is also a useful and effective procedure. The prevalence of colonoscopy use for CRC screening is increasing, and in the United States, colonoscopy was used in approximately 54% cases in 2019 [11]. However, the life-threatening adverse events of total colonoscopy should also be highlighted, including those related to the preparation stage. To perform CRC screening safely and efficiently, the development of novel CRC screening methods is required. Today, tumor biomarkers, such as mRNA, microRNA, cell-free DNA (cfDNA), and circulating tumor DNA (ctDNA), which are found in biological fluids, are receiving significant attention. A number of reports regarding the efficacy of liquid biopsy for CRC screening have been published; however, most of these reports described preliminary studies [12]. Artificial intelligence (AI) for the detection and characterization of colorectal tumor has been developed, and its efficacy has been reported. AI is expected to reduce the endoscopist’s burden and compensate for the differences in technical skill between endoscopists. 

Endoscopic submucosal resection (ESD) has become a standard endoscopic therapeutic method, and its use is widespread. However, the possibility of expanding the implementation of ESD is currently under discussion. To perform ESD safely, easily, and cost-effectively, efforts to further develop ESD should be continued. When considering the therapies that are best suited for colorectal tumors, occasionally, we encounter patients who remain indecisive regarding their treatment intervention. As reported in this Special Issue, Serra-Aracil et al. focused on cases of diagnostic uncertainty, such as the differentiation between stages T2 and T3 in cases of rectal adenomas that could possibly be adenocarcinomas, or between stages T1 and T2 in cases of rectal adenocarcinomas, because these differentiations might affect therapeutic strategy, transanal endoscopic surgery (TES), or total mesorectal excision. They analyzed 803 patients who underwent TES and concluded that TES can be recommended as an initial treatment for such patients. As they mentioned, a large-scale multicenter study is warranted [13]. Recently, robotic-assisted laparoscopic operations for rectal cancer have been developed and gained significant momentum [14]. A meta-analysis trial evaluated and confirmed the safety of robotic-assisted laparoscopic rectal surgery [15]. The outcomes of robotic-assisted laparoscopic rectal surgery showed that no significant changes in the 5-year overall survival rate, the 5-year disease-free survival rate, and local recurrence were observed in comparison to standard laparoscopic surgery [16].

Numerous novel drugs and procedures have been developed based on research and the clinical background of CRC, and their development has been remarkable. In the future, comprehensive genetic analysis is expected to lead to the realization of personalized medicine. 

This Special Issue focuses on the current status of, and challenges concerning, colorectal neoplasia in both its research and clinical aspects. Five valuable articles were published in this Special Issue. We believe that this Special Issue will help its readers in gaining knowledge of current information regarding both colorectal neoplasia research and clinical practice.
